# Anti-Aging Potential of Extracts from *Washingtonia filifera* Seeds

**DOI:** 10.3390/plants10010151

**Published:** 2021-01-14

**Authors:** Benedetta Era, Sonia Floris, Valeria Sogos, Clara Porcedda, Alessandra Piras, Rosaria Medda, Antonella Fais, Francesca Pintus

**Affiliations:** 1 Department of Life and Environmental Sciences, University of Cagliari, 09042 Monserrato, Cagliari, Italy; era@unica.it (B.E.); s.floris@unica.it (S.F.); rmedda@unica.it (R.M.); fpintus@unica.it (F.P.); 2 Department of Biomedical Sciences, University of Cagliari, 09042 Monserrato, Cagliari, Italy; sogos@unica.it (V.S.); porcedda.clara@gmail.com (C.P.); 3 Department of Chemical and Geological Sciences, University of Cagliari, 09042 Monserrato, Cagliari, Italy; apiras@unica.it

**Keywords:** enzyme inhibition, collagenase, elastase, tyrosinase, plant extracts, seeds, skin aging, *Washingtonia filifera*

## Abstract

The aim of this study was to test the inhibitory effect of fruit extracts from *Washingtonia filifera* on skin aging-related enzymes. The pulp extracts did not exert a significant enzyme inhibition while seed extracts from *W. filifera* exhibit anti-elastase, anti-collagenase, and anti-tyrosinase activities. Tyrosinase was mildly inhibited while a stronger effect was observed with respect to elastase and collagenase inhibition. Alcoholic extracts provided better results than aqueous extracts. Among them, methanol extracts showed the prominent enzyme inhibitory activities being IC_50_ value for elastase and collagenase comparable and even better than the reference compound. The inhibition mode of the most active extracts was investigated by Lineweaver-Burk plot analysis. Seed extracts from *W. filifera* were also investigated for their photo-protective effect by Mansur equation and the antioxidant activity of *W. filifera* extract was evaluated in oxidative-stressed cells. To evaluate the safety of the extract, the effect on cell viability of human keratinocytes cells was analyzed. Methanol extract presented the best photo-protective effect and exerted an antioxidant activity in a cellular system with no cytotoxic effect. The overall results demonstrate that *W. filifera* extracts are promising sources of bioactive compounds that could be used in cosmetic and pharmaceutical preparation.

## 1. Introduction

Skin aging is a complex biological phenomenon due to the physiological decrease in skin functions and several extrinsic environmental factors, such as UV radiation, chemicals, and reactive oxygen species (ROS). Skin is the largest and most exposed part of the body and exposure to solar UV radiation represents one of the most significant external stress-inducing factors: photo-induced skin aging associated with oxidative stress. ROS induced by UV irradiation can initiate complex molecular pathways, including the activation of enzymes that degrade extracellular matrix (ECM) proteins in the dermis, altering the skin integrity [1]. One of the main characteristics of skin aging is indeed the loss of structure of the ECM, which comprises numerous proteins, including collagen and elastin, all of which play a major role in retaining skin elasticity [2]. Degradation of ECM is mainly due to the enhanced activity of proteolytic enzymes, such as collagenase and elastase. Inhibition of these enzymatic activities by natural plant compounds might be a promising approach to prevent skin aging [3]. Collagenase (EC 3.4.24.3) belongs to the family of matrix metalloproteinases and it can degrade the triple-helical region of collagen under physiological conditions. Collagen is the fibrous component of the ECM and the major structural protein in human skin, providing structural support for bones, tendons, ligaments, and blood vessels. Elastase (EC 3.4.21.36) is a proteolytic enzyme involved in the physiological degradation of elastin, the ECM protein responsible for skin elasticity. An increase of elastase activity has been found in several diseases, for instance, psoriasis, dermatitis, inflammatory processes, and premature skin aging, which are closely associated with the formation of wrinkles [4].

Moreover, one of the major changes associated with wrinkling in the elderly is the appearance of the hyperpigmented spots, also known as senile lentigo or age spots. They are directly associated with uneven pigmentation due to the activity of another aging-related enzyme named tyrosinase. Tyrosinase (EC 1.14.18.1) is the rate-limiting enzyme in the metabolism of melanin. It catalyzes the hydroxylation of L-tyrosine to 3,4-dihydroxyphenylalanine (L-DOPA), followed by the oxidation of L-DOPA to dopaquinone. Oxidative polymerization of dopaquinone derivatives gives rise to melanin [5]. The synthesis of melanin pigments is a physiological process that plays a crucial role in preventing UV-induced skin damage by absorbing UV sunlight. Despite its advantages, excess production or abnormal accumulation of melanin causes skin problems such as the typical age spots. Since the skin is the most visible organ of the body, the premature appearance of wrinkles and hyperpigmentation can also cause emotional distress for some people.

Thus, inhibitors of all the above-described enzymes may represent increasingly important ingredients in cosmetics and medications to prevent skin aging [6].

Natural plant products could be a promising source of bioactive compounds. Of particular interest for anti-aging application are the extracts possessing multiple beneficial functions, such as the inhibition of aging-related enzymes and the capacity of scavenging free radicals.

**Figure 1:**
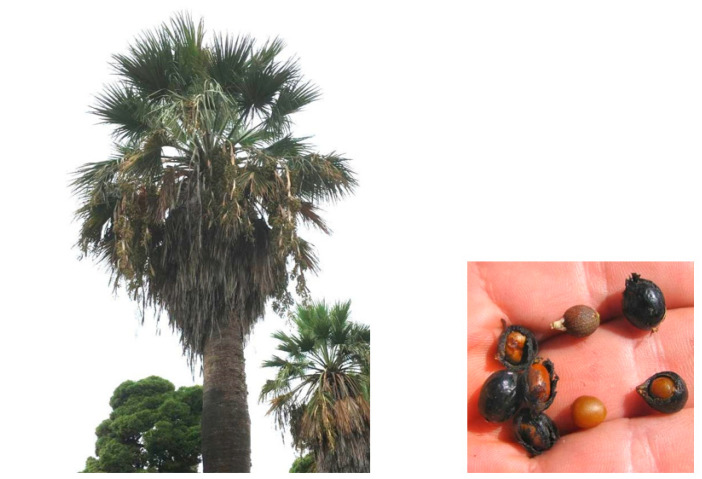
*Washingtonia filifera* (**left**) and its fruits (**right**), where pulp and seeds are well visible.

In our previous work, we described antioxidant capacity and several biological activities of *W. filifera* seed extracts [7]. *Washingtonia filifera* (Lindl.) H. Wendl., commonly known as the California fan palm or the desert fan palm, is an evergreen palm tree native to Southern California, Arizona, Mexico, and desert zones. This palm, with a height of 15–20 m, does not produce dates, but possesses sweet and tasty edible fruits. These berries have a very large, brown seed surrounded by a thin pulp (Figure 1).

*W. filifera* has been studied regarding, for example, its use as a potential source of novel cellulosic fibers [8] and the nutritional value of its fruits [9]; the phenolic composition and antioxidant activity of the aerial parts have been also reported [10]. In our previous study, we reported the good antioxidant activity of *W. filifera* seed extracts, which appeared to be a source of phenolic and flavonoid molecules [7]. The same extracts exerted an inhibitory effect on xanthine oxidase and cholinesterase enzymes, which represent key enzymes in the treatment of gout and Alzheimer’s disease, respectively.

The aim of this work was to extend the characterization of this plant and evaluate the potential use of its extracts as an anti-aging agent. Pulp and seed extracts were therefore investigated for their inhibitory activities toward elastase, collagenase, and tyrosinase activity, since they represent the key target enzymes for the prevention and treatment of skin photoaging. Moreover, the *W. filifera* extracts showing the best promising activities were analyzed for their in vitro cytotoxicity, cellular antioxidant activity, and photo-protective effects.

## 2. Results and Discussion

### 2.1. Enzyme Inhibition

*W. filifera* pulp and seed extracts were first tested at a concentration of 50 µg/mL. All the pulp extracts did not exert a significant enzyme inhibition (data not shown). The enzyme inhibitory activities of seed extracts were calculated and expressed as the half-maximal inhibitory concentration (IC_50_). Table 1 shows the IC_50_ values obtained from the seed extracts compared with those of the standard inhibitors, in order to evaluate the inhibitory strength of the samples.

As it could be observed, all the extracts weakly inhibit tyrosinase activity, with IC_50_ values higher than those of the standard, kojic acid. Better inhibition was observed against elastase and collagenase activities, and ethanolic (EEG and EES) and methanolic (MEG and MES) extracts exerted the best effects. The inhibitory activity of these samples against elastase was similar; the IC_50_ values ranged from 10.75 to 19.75 µg/mL and were comparable to that of the positive control (oleanolic acid; IC_50_ = 11.75 µg/mL).

Instead, collagenase activity was strongly inhibited by the extracts, which showed a higher potency than the standard epigallocatechin gallate, the IC_50_ being up to three-fold lower than the positive control. Considering that the extracts were a mixture of several compounds, the concentration of the single active molecules was even lower than the IC_50_ value, thus making the extracts even more promising sources of inhibitory compounds.

### 2.2. Kinetic Analysis by Lineweaver-Burk Plot

We focused our attention on ethanol and methanol extracts in order to investigate the mode of inhibition of these enzymes, since they had a better effect against elastase and collagenase activities. The kinetic of inhibition was determined by the Lineweaver–Burk double reciprocal plot. The assays were performed by increasing the concentration of the respective substrate in the absence and presence of the extracts at different concentrations.

**Figure 2:**
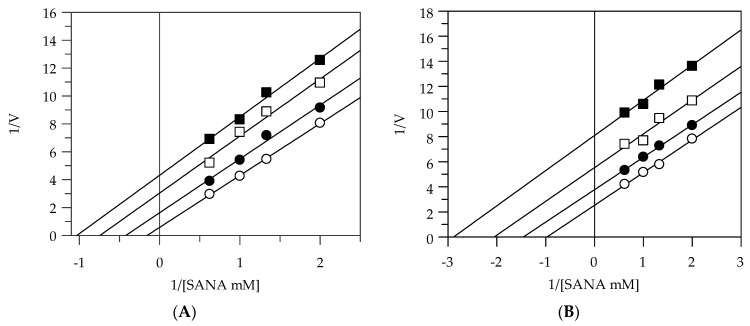

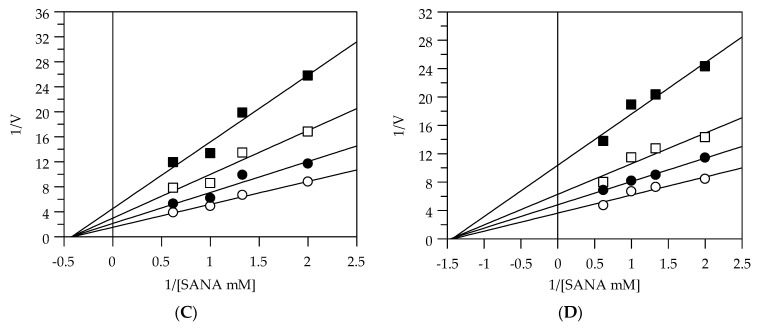
Inhibition of elastase activity, using N-succ-(Ala)3-nitroanilide (SANA) as a substrate. Lineweaver–Burk plots analysis of EEG (**A**), EES (**B**), MEG (**C**), and MES (**D**). Reaction conditions are reported in Section 3. The different concentrations of all the extracts were 0 (○), 5 (●), 10 (□), and 20 (■) µg/mL.

Table 2 shows that EEG and EES acted as uncompetitive inhibitors against elastase. In fact, the kinetic analysis of these extracts produced a family of parallel lines for increasing extract concentrations (Figure 2A,B). This kinetic analysis indicates that these extracts can bind with the enzyme–substrate complex. The equilibrium constant (K_IS_) was calculated from the replotting of the intercepts (1/V_max_) versus the inhibitor concentration, resulting in a value of 3.91 and 8.89 µg/mL for EEG and EES, respectively. The mode of inhibition of the methanolic extracts indeed revealed that these extracts act as a noncompetitive inhibitor. In fact, by increasing the concentration of extracts, a family of straight lines with different slopes, all intersecting on the abscissa, were found (Figure 2C,D). This analysis indicates that the extracts can bind not only to the enzyme–substrate complex but also to the free enzyme. The equilibrium constants for binding with the free enzyme (K_I_) and with the enzyme–substrate complex (K_IS_) were obtained either from the slope (K_m_/V_max_) or the 1/V_max_ values (𝑦-intercepts) plotted versus the inhibitor concentration, respectively.

**Figure 3:**
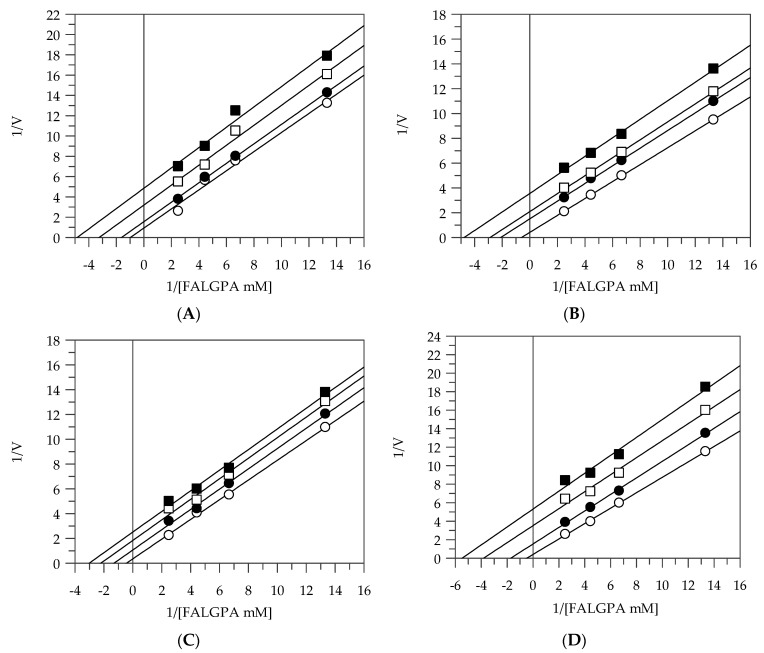
Inhibition of collagenase activity, using N-(3-[2-Furyl]-acryloyl)-Leu-Gly-Pro-Ala (FALGPA) as a substrate. Lineweaver–Burk plots analysis of EEG (**A**), EES (**B**), MEG (**C**), and MES (**D**). Reaction conditions are reported in Section 3. EEG and EES concentrations were 0 (○), 25 (●), 50 (□), and 75 (■) µg/mL; MEG and MES concentrations were 0 (○), 10 (●), 25 (□), and 50 (■) µg/mL.

Finally, the kinetic behavior of collagenase at different concentrations of extracts is shown in Figure 3A–D. All the extracts acted as uncompetitive inhibitors with K_IS_ values in the range of 7.58–13.04 µg/mL (Table 2).

In our previous work, we analyzed the alcoholic extracts of *W. filifera* seeds, using HPLC–DAD–ESI/MS. We have highlighted that the main phenolic compounds of these extracts consist of flavan-3-ol [7]. Among them, B-type procyanidins were the main compounds in the extracts of *W. filifera* seeds. A positive relationship between the degree of procyanidin polymerization and the capacity of the procyanidins to inhibit elastase was observed in previous paper [11,12]. Moreover, inhibitory activity against elastase and collagenase by some procyanidin compounds has been reported [13,14]. The synergic action of these compounds could contribute to explaining the significant inhibition of the *W. filifera* methanolic extract against both the enzymes.

### 2.3. Sun Protection Factor

Photoprotectant activity is very important for compounds with possible skin application, thus we determined the sun protection factor (SPF) of our extracts. The SPF indicates the ability of a substance to absorb UV rays, protecting the skin from the toxic effects produced by such radiation. Plant-based cosmetics have great potential in absorbing UV-radiation because plant extracts contain polyphenols, such as flavonoids or carotenoids. These compounds, having aromatic rings, can absorb UV rays and, therefore, can act as a sun filter. Since the alcoholic seed extracts of *W. filifera* contain phenolic and flavonoid compounds [7], the photo-protective effects of these extracts were evaluated. As shown in Table 3, all the analyzed extracts, at the concentration of 100 µg/mL, showed SPF values ranging from 1.52 to 3.35. Methanol extracts were revealed to possess the best photo-protective effects. UV rays are responsible for skin diseases and they trigger the processes that result in skin aging, oxidative stress, and wrinkle formation. Thus, reducing the absorption of this radiation enhances, in an indirect way, the antioxidant activities and the inhibition of aging-related enzymes.

### 2.4. Cell Viability and Intracellular ROS Level

Since oxidative stress is a key factor in causing aging and age-related damage, we also examined whether *W. filifera* extracts inhibited H_2_O_2_-induced ROS generation in a cellular system. In a previous paper, we described the antioxidant activities of seed extracts using a spectrophotometric method (ABTS assay) [7]. The samples were revealed to be a good source of phenolic compounds with antioxidant properties, with MEG showing the best activity. Since this extract had the best antioxidant activity and also great potential, considering the inhibitory activities against the enzyme tested (cholinesterase, xanthine oxidase, and the aging enzymes presented here), we decided to confirm the antioxidant capacity of MEG in a cellular model.

**Figure 4:**
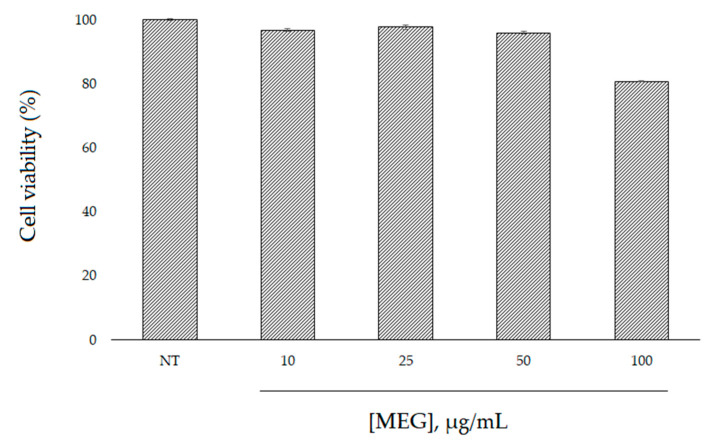
Effect of MEG on HaCaT cell viability. Cell viability was determined by an MTT assay after 24 h of incubation with the extract at different concentrations.

First, the effects of *W. filifera* extract on cell viability were investigated in HaCaT cells. The immortalized human keratinocytes HaCaT cell line has been extensively used as a model to study epidermal homeostasis [15]. In order to determine the safety of this extract, the cells were treated with various concentrations of the sample for 24 h and examined using an MTT test. The results indicate that the extract was not cytotoxic in HaCaT cells and only a small decrease (viability of 80%) was observed at 100 µg/mL (Figure 4).

**Figure 5:**
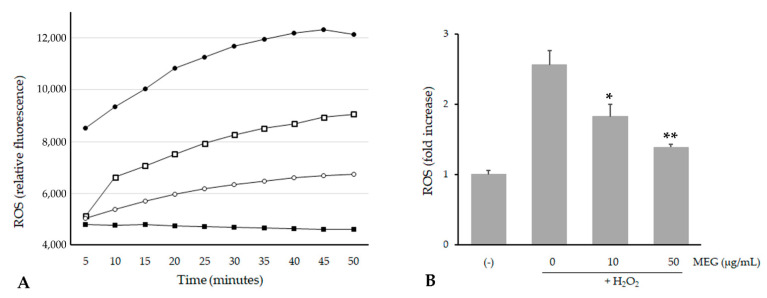
Inhibition of H_2_O_2_-induced reactive oxygen species (ROS) generation by MEG on HaCaT cells. (**A**) ROS levels (expressed as DCF fluorescence) in HaCaT cells pretreated with MEG and incubated with 1 mM of H_2_O_2_ up to 50 min. (■): untreated cells, (●): 1 mM H_2_O_2_, (□): 10 µg/mL MEG + H_2_O_2,_ (○): 50 µg/mL MEG + H_2_O_2_. (**B**) Effect of MEG on ROS production in HaCaT cells after a 30 min treatment with 1 mM H_2_O_2_. Data (means ± SE) are normalized to untreated controls. * *p* < 0.01 and ** *p* < 0.001 compared to the H_2_O_2_ treated group.

Since the viability was not affected until 50 µg/mL (viability of 96%), we decided to perform further cellular experimentation using up to this extract concentration. We evaluated ROS levels in the cells before and after oxidative stress, and after treatment with MEG. The study was conducted using 2′,7′-dichlorofluorescein diacetate (DCFH-DA), which easily diffuses through the cell membrane and is hydrolyzed by the endogenous esterases to DCFH. Rapid increases in DCF indicate the oxidation of DCFH by intracellular ROS, such as H_2_O_2_. As shown in Figure 5, H_2_O_2_ incubation significantly increased ROS formation in HaCaT cells, but treatment with the extract was able to inhibit H_2_O_2_-induced ROS production in a dose-response manner. Thus, these results confirm the antioxidant assays and suggest that MEG may also reduce the formation of ROS in cells.

The methanolic extract analyzed in this paper showed high antioxidant properties. The phenolic composition of this extract consisted of flavan-3-ol, and the B-type procyanidins were the main phenolic compounds [7]. Procyanidins, a group of polyphenolic bioflavonoids, have been reported to exhibit a wide range of biological, pharmacological, and chemoprotective properties against oxygen free radicals [16,17]. A previous study has shown that the anti-radical activity of procyanidins is strong at high concentrations [18]. Moreover, proanthocyanidin extracts are more effective superoxide radical-scavengers than antioxidant vitamin C and Trolox [19].

## 3. Materials and Methods

### 3.1. Chemicals

All chemical reagents were obtained as pure commercial products from Sigma Chemical Co. (St. Louis, MO, USA) unless otherwise indicated, and used without further purification.

### 3.2. Plant Sample Preparation

The fruits of *W. filifera* were collected in Tunisia in the areas of Gabès (G) and Sousse (S), and the plant materials were prepared according to the procedure previously described [7]. Pulp and seeds were separately lyophilized and then plant materials (25 g) were extracted in 100 mL of water (AE, aqueous extract), ethanol (EE, ethanol extract), or methanol (ME, methanol extract) for 72 h, at room temperature, in continuous stirring. After filtration and centrifugation at 10,000 rpm, aqueous extracts were then lyophilized, while the obtained ethanol and methanol extracts were concentrated under a vacuum, using a rotary evaporator for further analysis. Dried powers (1 mg/mL) were dissolved in DMSO before use.

### 3.3. Enzymatic Inhibition

The results of all the assays described below were expressed as a percentage of the blank control. Concentrations of extracts resulting in 50% inhibition of enzyme activity (IC_50_) were determined by interpolation of dose-response curves. The inhibition model was determined by performing assays at different concentrations of substrate and the absence and presence of the extracts at different concentrations. Kinetics data were analyzed using the Lineweaver–Burk plot. Data from the activity assays were recorded with an Ultrospec 2100 spectrophotometer (Biochrom Ltd., Cambridge, UK).

#### 3.3.1. Tyrosinase Inhibition Assay

The inhibition of tyrosinase activity by *W. filifera* extracts was determined by using 3,4-dihydroxyphenylalanine (L-DOPA) as a substrate [20]. The reaction mixture contained 25 mM phosphate buffer (pH 6.8), mushroom tyrosinase (100 U/mL, final concentration), with or without a plant extract solution. Then, L-DOPA (0.5 mM) was added into the mixture and the activity was determined by following the increase in absorbance at 492 nm, resulting from the formation of the dopachrome product. The concentration range of extract used for the mushroom tyrosinase inhibition assay was 0–0.3 mg/mL. In the assays performed without plant extracts, DMSO was added to the reaction mixture as a blank control. Kojic acid was used as a positive control.

#### 3.3.2. Elastase Inhibition Assay

Elastase inhibition was assayed monitoring the release of *p*-nitroaniline during cleavage of the substrate N-succ-(Ala)3-nitroanilide (SANA) by the action of the enzyme by the method described [21], with slight modifications. The assay was performed in 0.1 M Tris-HCl buffer (pH 8.0). Porcine pancreatic elastase (3.3 µg/mL) was incubated with or without the extract for 20 min and, after incubation, the substrate (1.6 mM) was added, and the enzyme activity was monitored at 410 nm. The control was performed with DMSO, while oleanolic acid was used as a positive control.

#### 3.3.3. Collagenase Inhibition Assay

Collagenase from Clostridium histolyticum was prepared in Tricine buffer 0.05 M, pH 7.5, containing 0.4 M NaCl and 0.01 M CaCl_2_, and incubated (1 U/mL) with test samples at different concentrations for 15 min. The synthetic substrate N-(3-[2-Furyl]-acryloyl)-Leu-Gly-Pro-Ala (FALGPA), prepared in the same buffer solution, was then added to start the reaction (with a final concentration of 0.8 mM). Absorbance was monitored at 340 nm [21]. The control was performed with DMSO, while epigallocatechin gallate was used as a positive control.

### 3.4. Determination of the In Vitro Sun Protection Factor

The sun protection factor of *W. filifera* extracts was determined using the UV absorbance method, according to the methodology described by Mansur et al. (1986) [22]. The absorbances of the extracts (0.1 mg/mL) were measured in the range of 290–320 nm, with 5 nm increments, and three determinations were made at each point. The SPF was calculated by applying the Mansur Equation:

SPF = CF × Σ^320^_290_ × EE(λ) × I(λ) × Abs(λ)

where CF = correction factor (10); EE (λ) = erythemogenic effect of radiation with wavelength λ; I(λ) = solar intensity spectrum; Abs(λ) = spectrophotometric absorbance values at wavelength λ. The values of EE(λ) × I(λ) are constant. They were determined by Sayre et al. (1979) [23] and are shown in Table 4.

### 3.5. Cell Culture and Intracellular ROS Levels

Human skin keratinocyte cell line HaCaT was cultured in Dulbecco’s Modified Eagle’s Medium (DMEM) containing 10% fetal bovine serum (FBS, Gibco, NY, USA) and 1% penicillin/streptomycin at 37 °C, in a humidified atmosphere, with 5% CO_2_. Cell viability was detected by the colorimetric 3-(4,5-dimethylthiazol-2-yl)-2,5-diphenyltetrazolium bromide (MTT) assay, as previously described, with minor modification [24]. After a 24 h incubation with MEG at different concentrations (0–100 µg/mL), the cells were labeled with MTT solution for 3 h at 37 °C. The resulting violet formazan precipitates were dissolved in DMSO and the absorbance of each well was determined at 560 nm using a microplate reader with a 630 nm reference.

The cellular ROS levels were determined with the DCFH-DA method [25]. HaCaT cells were treated with various concentrations of MEG (0–50 µg/mL) for 24 h. Then, the cells were incubated with DCFH-DA (10 µM) at 37 °C for 30 min. After incubation, 1 mM H_2_O_2_ was added to the wells, and the fluorescence intensity of DCF was immediately measured using a fluorescent plate reader at an excitation wavelength of 485 nm and an emission wavelength of 530 nm, taking readings at intervals of 5 min for 50 min.

### 3.6. Data Analysis

All experiments were performed in triplicates and the data were expressed as mean ± standard deviation (SD). Statistical differences were evaluated using GraphPad Prism software version 8 (San Diego, CA, USA). Comparison between groups was conducted by one-way analysis of variance (ANOVA) followed by the Tukey Multiple Comparisons Test. A *p*-value of less than 0.05 was considered statistically significant.

## 4. Conclusions

**Figure 6:**
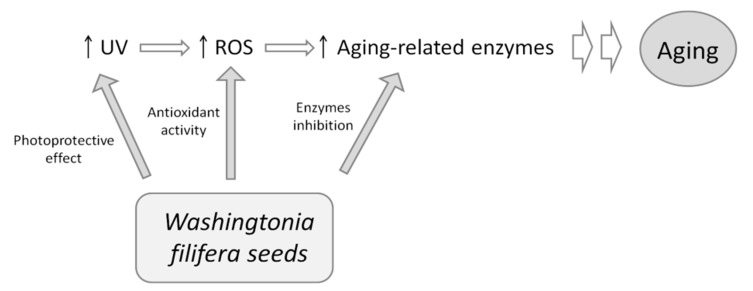
Effect of *W. filifera* seed extracts on aging process.

In conclusion, we report, for the first time, that seed extracts from *W. filifera* are effective in inhibiting the key enzymes implicated in skin aging. Natural or “intrinsic” aging is a physiological phenomenon that occurs in all human tissue as a consequence of the passage of time. However, skin is also exposed to external stress-inducing factors that represent a major cause of premature skin aging. “Extrinsic” aging is mainly related to UV-induced damage of the connective tissue of the skin. UV rays cause oxidative stress, which is responsible for the activation of the enzymes degrading the ECM, and the appearance of wrinkles and age spots. This manuscript reports the importance that seeds of *W. filifera* could have in this context. As shown in the Figure 6, extracts could act in the prevention of premature aging, acting simultaneously on several fronts.

First, the extracts can act at the beginning of the process via their photoprotective effects and, therefore, reduce UV ray absorption. Then, they showed a good antioxidant effect with methanol extract, preventing ROS formation in a cellular system, without cell toxicity. Finally, all the extracts, in particular, the methanolic samples, could inhibit collagenase, elastase, and tyrosinase (the latter to a minor extent). Overall, the results obtained show that *W. filifera* could be a source of bioactive molecules and encourage further experiments in order to isolate the single active components responsible for the observed activities.

## Figures and Tables

**Table 1:** Inhibition of the aqueous (AE), ethanolic (EE), and methanolic (ME) extracts from *W. filifera* collected in the areas of Sousse (S) and Gabès (G), expressed as IC_50_ values (µg/mL). Standard compounds are kojic acid, oleanolic acid, and epigallocatechin gallate for tyrosinase, elastase, and collagenase, respectively.

Plant Extracts	IC_50_ (µg/mL)		
	Tyrosinase *	Elastase *	Collagenase *
EEG	73.0 ± 5.09 ^a^	17.69 ± 2.81 ^a^	55.2 ± 19.09 ^a^
EES	89.0 ± 3.60 ^b^	19.75 ± 5.55 ^a^	50.04 ± 6.87 ^a^
MEG	89.5 ± 4.35 ^b^	10.76 ± 3.38 ^a^	50.03 ± 1.18 ^a^
MES	139.0 ± 3.34 ^c^	12.47 ± 3.11 ^a^	33.36 ± 13.06 ^a^
AEG	90.0 ± 2.11 ^b^	70.1 ± 4.56 ^b^	ND
AES	70.0 ± 3.17 ^a^	47.66 ± 2.88 ^c^	ND
Kojic acid	17.9 ± 0.98 ^d^	-	-
Oleanolic acid	-	11.75 ± 0.63 ^a^	-
Epigallocatechin gallate	-	-	120.8 ± 6.22 ^b^

Each value is the mean ± SD of three independent measurements (*n* = 3). * Different letters within the same column denote statistically significant differences between extracts (*p* < 0.05).

**Table 2:** Kinetic parameters of elastase and collagenase inhibition by *W. filifera* extracts.

Elastase			
Plant Extracts	Inhibition Type	K_I_ (µg/mL)	K_IS_ (µg/mL)
EEG	uncompetitive	-	3.91
EES	uncompetitive	-	8.89
MEG	noncompetitive	9.66	9.55
MES	noncompetitive	9.48	9.57
**Collagenase**			
EEG	uncompetitive	-	11.49
EES	uncompetitive	-	9.64
MEG	uncompetitive	-	13.04
MES	uncompetitive	-	7.58

**Table 3:** Absorbance and sun protection factor (SPF) values of ethanolic and methanolic extracts of *W. filifera*.

Wavelength (nm)	Absorbance			
	EEG	EES	MEG	MES
290	0.377	0.349	0.585	0.829
295	0.202	0.19	0.31	0.445
300	0.156	0.146	0.237	0.345
305	0.145	0.137	0.218	0.319
310	0.135	0.128	0.203	0.298
315	0.123	0.116	0.183	0.269
320	0.108	0.103	0.16	0.238
SPF	1.52	1.43	2.30	3.35

**Table 4:** E (λ) and I (λ) values used for SPF calculation.

Wavelength (nm)	EE × I
290	0.0150
295	0.0817
300	0.2874
305	0.3278
310	0.1864
315	0.0837
320	0.0180
